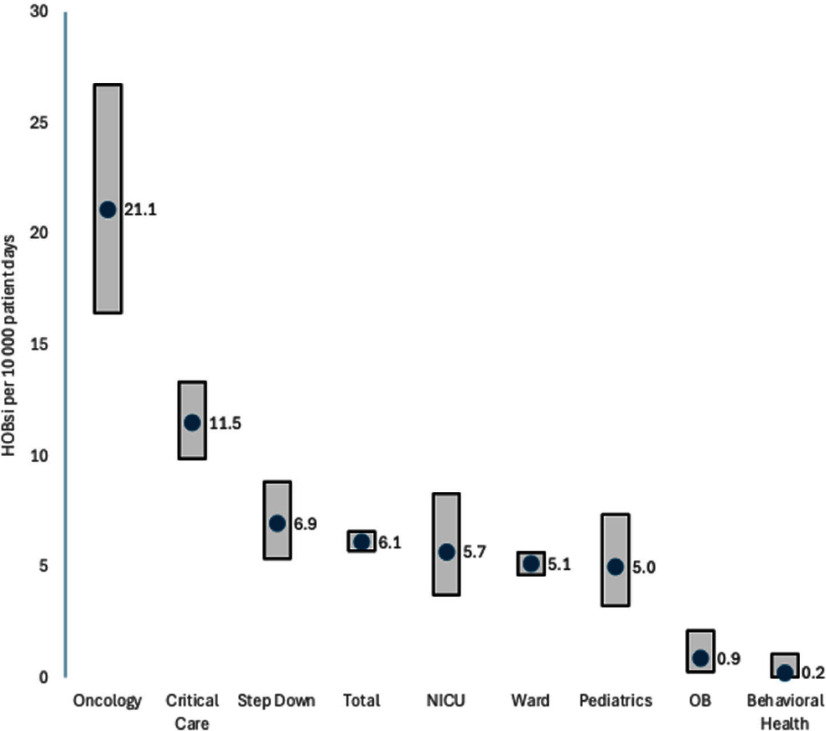# Hospital-Onset Bloodstream Infection Varies by Hospital Location Type

**DOI:** 10.1017/ash.2025.297

**Published:** 2025-09-24

**Authors:** Pragya Dhaubhadel, Prasanthi Limgala, Heather Stafford, Mark Shelly

**Affiliations:** 1 Geisinger; 2 Geisinger; 3 Geisinger Health System; 4 Geisinger

## Abstract

**Objectives:** To characterize the incidence and contributing factors related to hospital-onset bloodstream infection (HOBsi) in a nine hospital healthcare system. **Background:** Bloodstream infections that develop during hospitalization are critical measures of healthcare quality. Though these events are measured in part through CMS reports of central line-associated bloodstream infections (CLABSIs) and MRSA bloodstream infections. A newer metric has been introduced by National Healthcare Safety Network (NHSN) to measure any case of bloodstream infection with onset on or after hospital day four. There is no established benchmark rate for HOBsi and its clinical understanding remains complex. **Methods:** Positive blood cultures obtained on or after hospital day four from nine hospitals across northeast and central Pennsylvania were included in this study, spanning July 2021 to June 2024. Cases were classified based on NHSN criteria: primary bloodstream infections (BSIs), CLABSIs, mucosal barrier injury-related infections, and secondary bacteremia with identified sources (e.g., pneumonia, urinary tract infections, gastrointestinal infection or surgical site infection). **Results:** A total of 739 HOBsi cases occurred in 1,186,510 patient days over three years, for a rate of 6.13 (95% confidence interval 5.69 to 6.59). The rates varied significantly by hospital unit type (p=0.002) (Figure). Oncology wards had the highest HOB rate (21.1 infections per 10,000 patient days), followed by critical care units at 11.5. Behavioral health and obstetric wards had the lowest HOB rates. When location type was considered, the rates between hospital campuses were not significantly different. In multivariate regression, the central-line device use ratio further influenced the HOBsi rate (p=0.002). Primary BSIs accounted for 49.3% of cases, while 22.1% met the criteria for CLABSI. When NHSN-defined source was found (secondary BSIs), pneumonia was the most common source (6.5%), followed by urinary tract infections (5.5%), gastrointestinal tract infections (3.5%), surgical site infections (3%), and other sources (6%). Mucosal barrier injury-related HOBsi comprised 4.2% of cases. **Conclusion:** This quality measure significantly expands the scope of infection events over CLABSI. HOBsi is closely associated with the hospital location type. Device use may further stratify for severity. This study establishes some initial benchmarks. Understanding the likely source of bacteremia will be important in finding ways to target strategies to reduce HOBsi.

**Figure:**